# Biocompatibility of the Biopolymer Cyanoflan for Applications in Skin Wound Healing

**DOI:** 10.3390/md19030147

**Published:** 2021-03-11

**Authors:** Raquel Costa, Luís Costa, Ilda Rodrigues, Catarina Meireles, Raquel Soares, Paula Tamagnini, Rita Mota

**Affiliations:** 1 i3S-Instituto de Investigação e Inovação em Saúde, Universidade do Porto, Rua Alfredo Allen, 208, 4200-135 Porto, Portugal; mrcosta@med.up.pt (R.C.); luiscostabioeng@gmail.com (L.C.); catarina.meireles@i3s.up.pt (C.M.); raqsoa@med.up.pt (R.S.); pmtamagn@ibmc.up.pt (P.T.); 2 Unit of Biochemistry, Department of Biomedicine, Faculty of Medicine, University of Porto, Al. Prof. Hernâni Monteiro, 4200-319 Porto, Portugal; irodrigues@med.up.pt; 3 IBMC-Instituto de Biologia Molecular e Celular, Universidade do Porto, Rua Alfredo Allen, 208, 4200-135 Porto, Portugal; 4 Departamento de Biologia, Faculdade de Ciências, Universidade do Porto, Rua do Campo Alegre, Edifício FC4, 4169-007 Porto, Portugal

**Keywords:** antioxidant, cyanobacteria, Cyanoflan, wound healing, wound dressings, skin regeneration

## Abstract

There is a great demand for the development of novel wound dressings to overcome the time and costs of wound care performed by a vast number of clinicians, especially in the current overburdened healthcare systems. In this study, Cyanoflan, a biopolymer secreted by a marine unicellular cyanobacterium, was evaluated as a potential biomaterial for wound healing. Cyanoflan effects on cell viability, apoptosis, and migration were assessed in vitro, while the effect on tissue regeneration and biosafety was evaluated in healthy Wistar rats. The cell viability and apoptosis of fibroblasts and endothelial cells was not influenced by the treatment with different concentrations of Cyanoflan, as observed by flow cytometry. Moreover, the presence of Cyanoflan did not affect cell motility and migratory capacity, nor did it induce reactive oxygen species production, even revealing an antioxidant behavior regarding the endothelial cells. Furthermore, the skin wound healing in vivo assay demonstrated that Cyanoflan perfectly adapted to the wound bed without inducing systemic or local oxidative or inflammatory reaction. Altogether, these results suggest that Cyanoflan is a promising biopolymer for the development of innovative applications to overcome the many challenges that still exist in skin wound healing.

## 1. Introduction

Wound healing is a complex and highly regulated process that is critical to restore the barrier function of the skin. However, this process can fail due to several reasons, such as repeated tissue abrasions, poor primary treatment or patient-related health problems (e.g., diabetes or obesity), resulting in a significant burden to the patients and medical systems [1]. Therefore, distinct wound dressings are being developed aiming at restricting liquid and microbial penetration but keeping the exchange of air and water vapor and being easy to remove without damaging the wounded tissue [2]. The dressings developed during the last years include transparent film dressings, hydrogels, hydrophilic foams, hydrocolloids, alginates and antibacterial and biologic dressings [3]. However, the efficiency of the dressing is highly dependent on the type of wound.

Within the materials that can be used to produce dressings, biopolymers are advantageous in comparison to synthetic ones due to their biocompatibility, biodegradability and lower antigenicity, and because they are derived from renewable natural sources [4]. In addition, the native properties and diverse composition of biopolymers allow them to be easily tunable into hydrogels or scaffolds and combined with drugs or other polymers, as well as to absorb large volumes of water when in dry state and donate water when hydrated [5]. These biopolymers are usually repeated units of polysaccharides or peptides, and the mostly used are collagen, cellulose, chitosan, alginate, hyaluronan, fucoidan and carrageenan [4]. In this context, despite their promising features, such as anionic nature and intrinsic bioactivity, the polysaccharidic biopolymers produced by cyanobacteria and microalgae are still unexplored natural resources in wound healing.

Cyanoflan is a biopolymer secreted by the marine unicellular cyanobacterium *Crocosphaera chwakensis* CCY0110 (previously known as *Cyanothece* sp. CCY 0110). An extensive characterization revealed the high structural complexity of the Cyanoflan, which is composed by nine different monosaccharide residues, including two uronic acids, sulfate groups and peptides [6]. Moreover, Cyanoflan is easy to isolate and recover since the majority of the polymer is released by the cyanobacterium to the culture medium, and the costs are reduced due to the minimal nutrient requirements of cyanobacteria (ability to sequester carbon dioxide). In addition, several studies have been describing the versatility of this biopolymer for biomedicine. For example, Cyanoflan can be used as a vehicle for the controlled delivery of functional proteins or vitamins [7,8], to develop infection-preventive anti-adhesive coatings [9,10], or even be used as thickener, texturizing, gelling and emulsion-stabilizing agent [6].

The present study aimed at evaluating the potential of Cyanoflan as a biomaterial for the development of novel wound dressings to promote wound healing. For this purpose, the effect of the biopolymer in fibroblasts and endothelial cells’ viability, apoptosis and migration in vitro was assessed. In addition, a skin wound healing in vivo assay was performed in healthy Wistar rats to evaluate Cyanoflan biosafety and its effect on tissue regeneration.

## 2. Results and Discussion

### 2.1. Cyanoflan Effect on Cell Viability or Apoptosis in Human Dermal Cell Lines

**Figure 1:**
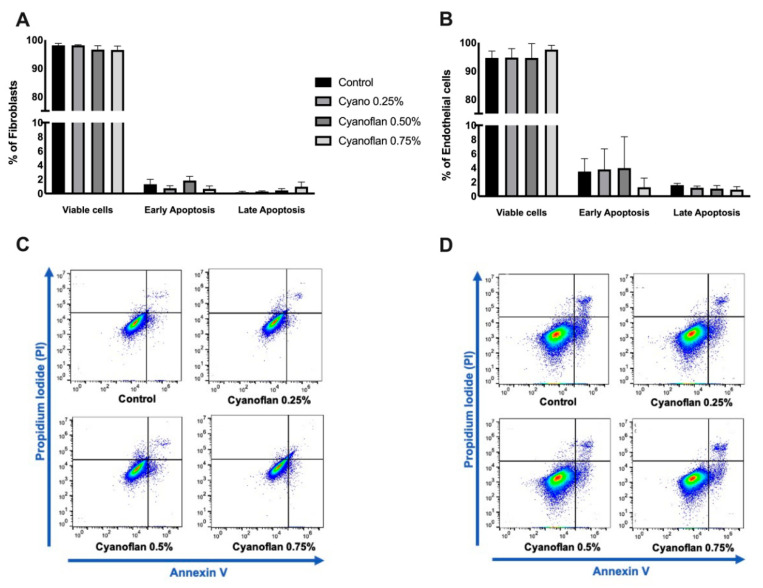
Flow cytometry analysis of viability and cell death in (**A**) human dermal fibroblasts (HDF) and (**B**) human microvascular endothelial cells (HMEC-1), using Annexin V-Fluorescein isothiocyanate/Propidium Iodide staining. Cells were treated with Cyanoflan at 0.25%, 0.5%, 0.75% *(w*/*v)* or control (incomplete cell culture medium), for 24 h. (**C**,**D**) Representative plots of three independent experiments are shown. Results are represented as percentage of viable cells and cells in early or late apoptosis and expressed as mean ± Standard Error of Mean (SEM).

Prior to in vivo application, the cytotoxic and proliferative effects of wound dressings need to be evaluated in vitro, with the biocompatibility being one of the most important features during the development of novel wound dressings. As the skin wounds are exposed to potential toxic and infectious environments, it is important to ensure that the materials used in wound dressings production would not exacerbate the healing process, being themselves toxic [11]. To evaluate the potential cytotoxic activity of the biopolymer Cyanoflan, flow cytometry analysis with Propidium Iodide/Annexin V double staining was performed in human dermal fibroblasts (HDF) and human microvascular endothelial cells (HMEC-1). Both cell types were treated during 24 h with Cyanoflan at concentrations of 0.25%, 0.50% and 0.75% (*w*/*v*) diluted in incomplete cell culture medium or left untreated (control group). Our results, depicted in Figure 1, show that Cyanoflan did not have cytotoxic effects on these cells. Actually, exposed cells exhibit similar levels of viability and apoptosis rate compared to the control group (94.63% ± 2.41% of viable cells and 5.03% ± 2.07% of cells in early or late apoptosis for HMEC-1, and 98.13% ± 0.64% of viable cells and 2.19% ± 0.66% of cells in early or late apoptosis for HDF). Since the biocompatibility of Cyanoflan is similar with the three concentrations tested, the following studies were carried out with the two lower doses of the biopolymer since their viscosity was easier to handle.

### 2.2. Cyanoflan Effect on Injury: In Vitro Assay 

**Figure 2:**
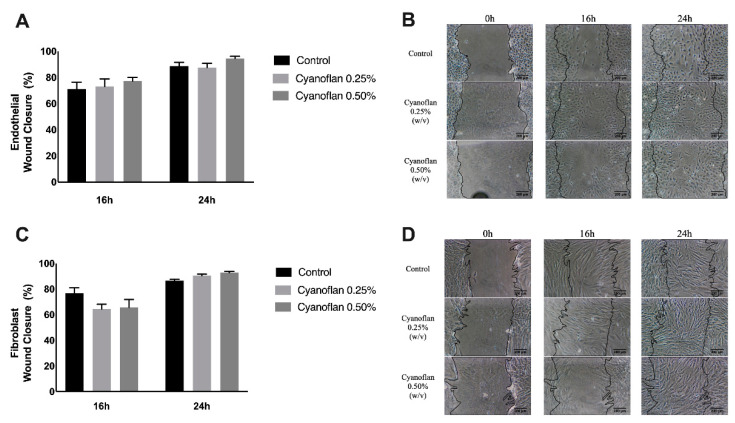
Cell migration analyzed by an injury assay. (**A**,**B**) Migratory capacity of human microvascular endothelial cells and (**C**,**D**) human dermal fibroblasts, after treatment with Cyanoflan at 0.25%, 0.5% (*w*/*v*) or control (incomplete cell culture medium), for 16 and 24 h. Results are represented as percentage of control group, expressed as mean ± SEM of three independent experiments. Scale bars = 200 μm (**B**,**D**).

When wound dressings are directly applied to a chronic wound injury, interactions between the cells and dressing will dictate cell fate by possible modifications of the cell metabolism, proliferation capacity or motility function. An injury assay was conducted to evaluate endothelial and fibroblast cell migration, allowing the visualization of cell motility and direction of cell movement at distinct timepoints and the morphological characteristics of the cells, such as lamellipodium formation and tail retraction [12]. The injury was inflicted, the cells were exposed to different treatments and the effects were monitored and quantified at 16 and 24 h (Figure 2). The closure rate was similar for both HMEC-1 (Figure 2A,B) and HDF (Figure 2C,D) cells that were able to migrate and proliferate towards the acellular area, at similar levels in comparison to the control group. After 24 h, the wounds are almost completely closed. No significant differences were observed between the groups, suggesting that Cyanoflan does not affect cell motility and migratory capacity. After 24 h, both HMEC-1 and HDF cells were able to fill the injured area at 87.61% ± 3.30% and 90.47% ± 1.24% respectively, for Cyanoflan 0.25% (*w*/*v*), and 94.52% ± 1.62% and 92.80% ± 1.05% respectively, for Cyanoflan 0.5% (*w*/*v*). These migration levels were similar when compared to the control group, in which HMEC-1 and HDF migrated 88.66% ± 2.82% and 86.52% ± 1.11%, respectively. These results suggest that, in accordance with the cytotoxic and the proliferative results (see below), Cyanoflan at 0.25% and 0.5% *(w*/*v)* allows cells to maintain its physiological morphology and function, as expected based on published results using other natural polymers [13]. Fibroblast and endothelial cell migration is one of the main bottlenecks during wound healing since it affects crucial processes, namely fibrinolysis, the formation of new extracellular matrix and collagen fibers to guide and support the other skin cells involved in the regeneration and remodeling, to ensure an effective wound healing and wound closure. In contrast, a study conducted by Alvarez et al., with human dermal fibroblasts exposed to cyanobacterial polymers released by *Nostoc* spp., showed faster kinetics of wound closure during the in vitro experiment by encouraging fibroblast migration to the wound site [14]. 

### 2.3. Reactive Oxygen Species (ROS) Production Assessment

**Figure 3:**
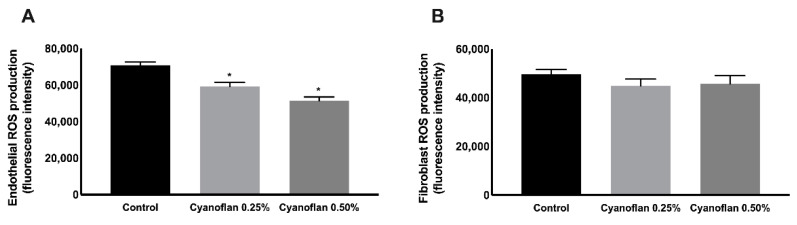
Quantification of reactive oxygen species (ROS) production by 2′,7′–dichlorofluorescin diacetate (DCFDA) fluorescence detection in (**A**) human microvascular endothelial cells and (**B**) human dermal fibroblasts, after treatment with Cyanoflan at 0.25%, 0.5% (*w*/*v*) or control (incomplete cell culture medium). Results are represented as mean of fluorescence units ± SEM of three independent experiments (* *p*-value ≤ 0.05).

After HMEC-1 and HDF exposure to Cyanoflan 0.25% and 0.5% (*w*/*v*), the quantification of ROS production was assessed (Figure 3) to uncover if the applied treatments would induce an oxidative effect in vitro. Interestingly, our results suggest that Cyanoflan not only did not induce ROS production, but also displayed a dose-dependent antioxidant behavior in endothelial cells compared to the control group (Figure 3A). A reduction from 70,908 ± 1739 fluorescence units for the control group to 59,206 ± 2249 for Cyanoflan 0.25% (*p* = 0.009) was observed, and to 51,351 ± 2132 for Cyanoflan 0.5% (*p* = 0.0003). Regarding fibroblasts, the production of ROS is similar among the different groups (Figure 3B), with the fluorescence intensity of 49,653 ± 1912 for the control group, and 44,802 ± 2875 and 45,660 ± 3413 for Cyanoflan 0.25% and 0.5% (*w*/*v*), respectively. Several studies have correlated biopolymers’ antioxidant activity with the metal chelating capacity [14,15,16]. This may explain the result obtained with Cyanoflan, since it was already reported that this biopolymer is an efficient metal biosorbent [17]. The antioxidant activity, namely through the reduction of ROS production, has also been described for several cyanobacterial polymers, e.g., for Nostoglycan, the polysaccharide produced by the colonial filamentous cyanobacterium *Nostoc sphaeroides* [18]. Since wound healing is severely hampered by the excessive production of ROS, the metal chelation and ROS scavenging abilities demonstrated by our and others cyanobacterial polymers could reduce ROS levels at the wound site and consequently prevent tissue damage.

Overall, in vitro data suggest that Cyanoflan application is safe and suitable for skin cells, namely endothelial cells and fibroblasts. Since Cyanoflan have demonstrated biocompatibility to these cells, which are key players during the wound healing process and tissue regeneration, Cyanoflan was further evaluated in vivo, using a skin wound healing assay to validate its potential application for wound care.

### 2.4. Bioactivity and Safety Application of Cyanoflan In Vivo

**Figure 4:**
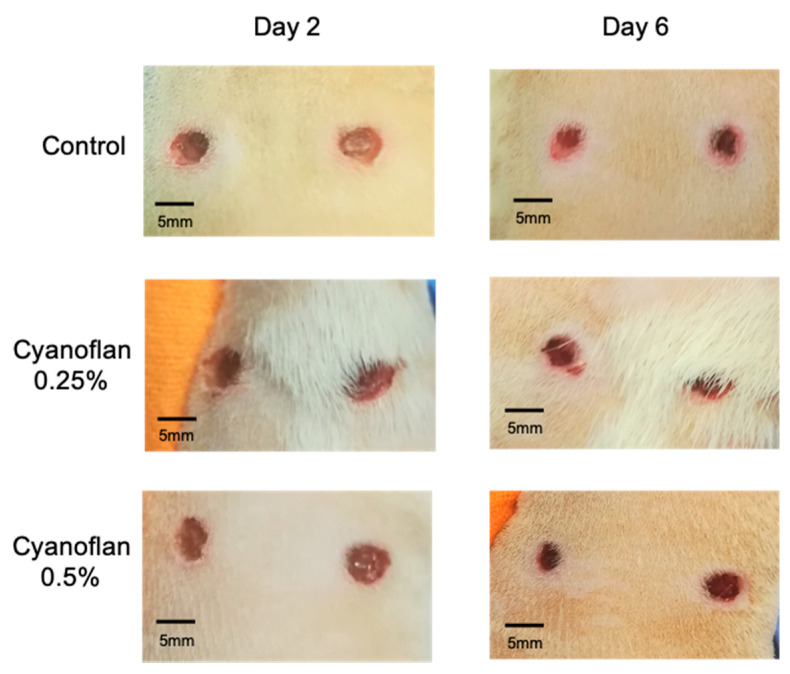
Cyanoflan effect on wound closure, over the 7 days of the skin wound healing assay. Cyanoflan at 0.25% and 0.5% *(w*/*v)* or control (saline solution) were applied to 3 cm-diameter full-thickness excisional wounds at days 2 and 6 post-injury.

To evaluate the effect on tissue regeneration and biosafety of Cyanoflan in vivo, a skin wound healing assay was performed in healthy Wistar rats. To avoid using synthetic dressings to cover the wound, Cyanoflan was reticulated with calcium chloride, an ionic crosslinker frequently used for the rapid gelation of biopolymers [8,19]. The treatment was applied and was able to fill the entire wound bed, without overflowing to the surrounding tissue, being absorbed and reapplied every 2 days. Wound scars of the Cyanoflan-treated groups formed more quickly and increased in size compared to the control group, especially when the concentration of 0.5% (*w*/*v*) was used (Figure 4).

**Figure 5:**
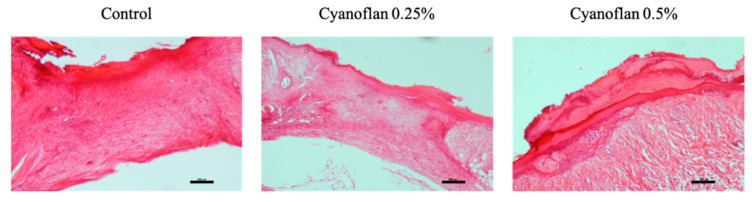
Representative images of histological analyses after Cyanoflan treatment in a rat model of skin wound healing assay, with hematoxylin and eosin staining. Each wound was treated with Cyanoflan at 0.25%, 0.5% (*w*/*v*) or saline solution (control). Magnification of 10×. Scale bars = 200 μm.

Regarding the wound closure, our results showed that during the experimental period, wounds had a slight decrease every two days in both groups treated with Cyanoflan and the one treated with saline solution (control), reaching a similar size at day 7. Consequently, the wound healing process in rats treated or not with Cyanoflan did not exhibit visual differences regarding wound closure rate (Figure 4). However, the histological analysis revealed that the wounds treated with Cyanoflan 0.25% exhibit less extension of inflammatory infiltration and present a reduction in wound area, when compared to the control group (Figure 5). Furthermore, there was a dose-dependent effect with Cyanoflan 0.5% with the formation of more scar tissue and reduced wound depth, and a better resolution of the inflammatory phase of the wound healing process (with reduction of inflammatory cells and extension of granulation tissue), suggesting that this formulation could further improve the healing progression.

**Figure 6:**
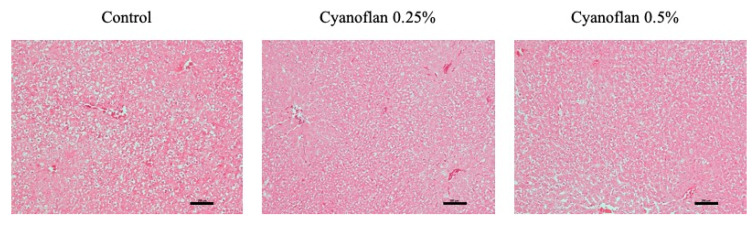
Representative images of histological analyses of liver, using hematoxylin and eosin staining. Rats were topically treated with Cyanoflan at 0.25%, 0.5% (*w*/*v*) or saline solution (control), in a skin wound healing assay. Magnification of 40×. Scale bars = 200 μm.

Since the topical application of Cyanoflan at 0.25% and 0.5% (*w*/*v*) concentrations was performed every two days, it is important to discard any possible local or systemic toxicity. To validate this, some inflammatory mediators were measured on the skin tissue and blood, and a liver histological evaluation was also performed. As depicted in Figure 6, liver histology is similar for all experimental conditions, excluding hepatotoxic behavior of Cyanoflan.

**Figure 7:**
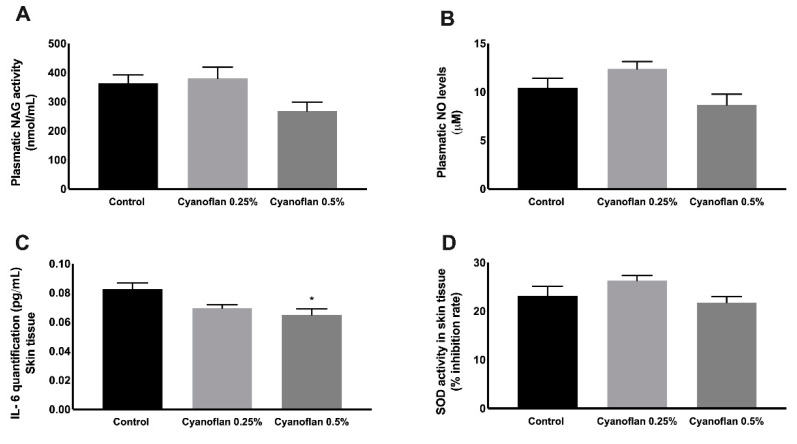
Quantification of inflammatory and antioxidant defense mediators. (**A**) Quantification of N-acetyl-ß-D-glucosaminidase (NAG) plasmatic activity assessed by enzymatic assays, (**B**) assessment of plasmatic nitric oxide (NO) levels by enzymatic assay, (**C**) quantification of skin levels of Interleukin-6 by Enzyme-Linked Immunosorbent assay (ELISA), and (**D**) Superoxide dismutase (SOD) activity determination by enzymatic assay. Results are expressed as mean ± SEM (*n* = 6) (* *p*-value ≤ 0.05).

Regarding systemic inflammatory markers, the activity of N-acetyl-ß-D-glucosaminidase (NAG), an enzyme secreted by activated macrophages, and the levels of nitric oxide (NO) were quantified in rats’ plasma (Figure 7A,B). A slight increase in NAG and NO levels was observed for Cyanoflan 0.25% (380.50 ± 38.31 nmol/mL and 12.37 ± 0.77 μM, respectively), and a small decrease for Cyanoflan 0.5% (268.20 ± 30.37 nmol/mL for NAG and 8.66 ± 1.11 μM for NO), when compared to the control group (363.30 ± 28.73 nmol/mL for NAG and 10.42 ± 1.00 μM for NO), without reaching statistical significance.

Additionally, pro-inflammatory cytokine Interleukin-6 was quantified in skin lysates, as well as the enzymatic antioxidant endogenous system, Superoxide dismutase. As depicted in Figure 7C, the application of Cyanoflan did not trigger a local inflammatory reaction. In contrast, our results suggest that there was a reduction on local inflammation due to Cyanoflan application at 0.5% (*w*/*v*) to interleukin-6 levels of 0.065 ± 0.004 pg/mL compared to 0.08 ± 0.004 pg/mL for the control group (*p =* 0.01). During the wound healing process, neutrophils and macrophages produce high amounts of ROS at the wound site as a defense against invading microorganisms. Additionally, other cells, namely fibroblasts, are also able to produce ROS, when stimulated by pro-inflammatory cytokines [20]. However, ROS could also be produced in response to toxic molecules, which could be harmful due to the sensitivity of some cellular components to oxidation, leading to cell death and tissue damage [20]. Based on our results in vitro, we did not expect to have increased levels of ROS in response to Cyanoflan treatments, but we performed a quantification of SOD activity to clarify if the application of the polymer could somehow interfere with the mechanisms that protect organisms against oxidative stress. According to Figure 7D, the SOD inhibition rate had a small increase for Cyanoflan 0.25% (26.32% ± 1.01%) and a slight decrease for Cyanoflan 0.5% (21.78% ± 1.23%), when compared to the control group (23.13% ± 1.99%), without reaching statistical significance.

Only a few cyanobacterial polymers have been studied envisaging the skin protection and/or regeneration. The exopolymers isolated from the strains *Tolypothrix tenuis* and *Anabaena* spp. were described as potential biomaterials for wound repair as a result of their antioxidant behavior due to their antiradical potential and iron chelating activity. In addition, these polymers exhibited hemostatic activity through strong thrombogenicity and shorter coagulation time [15,21]. Another example is Sacran, the polymer extracted from *Aphanothece sacrum,* which has the potential to reduce atopic dermatitis by downregulating inflammatory mediators in stimulated macrophages, and a similar result was obtained in a clinical study with 25 patients with atopic dermatitis, ameliorating skin condition [22]. More recently, hydrogels produced with polymers released by *Nostoc* spp. exhibited promising properties for the treatment of skin injuries through its potential to promote fibroblast proliferation and migration in vitro, crucial to assist in the repair and re-modelling of damaged skin [14]. Taking into account these emerging data, cyanobacterial polymers are promising novel biomaterials for skin regeneration (either by themselves or functionalized). In addition, the amount produced by our particular cyanobacterial strain combined with the straightforward isolation procedure of Cyanoflan (secreted to the culture medium) and its native features (e.g., high molecular mass and anionic character) contribute to facilitate the development of a cost-effective solution for wound healing.

## 3. Materials and Methods 

### 3.1. Cyanobacterium Growth Conditions and Cyanoflan Isolation

The unicellular cyanobacterium *Crocosphaera chwakensis* CCY0110 (previously *Cyanothece* sp. CCY 0110; Culture Collection of Yerseke, The Netherlands) was grown in 2 L bioreactors with Artificial Seawater (ASNIII) medium [23], at 25 °C, under a 16 h light (30 µE/m^2^/s)/8 h dark regimen, with orbital stirring (150 rpm) and aeration (1.2 L/min), until an optical density (OD730 nm) of ≈ 3–4. The isolation of the extracellular biopolymer named Cyanoflan was then performed according to Reference [9]. Briefly, the culture was dialyzed (12–14 kDa cut-off) for 48 h and then the cells were removed by high-speed centrifugation and the supernatant was precipitated twice with two volumes of 99% ethanol at 4 °C overnight. The collected Cyanoflan was lyophilized, grounded, and stored in a desiccator until further steps.

### 3.2. Cell Culture Experiments

Human microvascular endothelial cells (HMEC-1, ATCC, Barcelona, Spain) were cultured in Roswell Park Memorial Institute (RPMI) 1640 medium (Life Technologies, Carlsbad, CA, USA) supplemented with 10% (*v*/*v*) Fetal Bovine Serum (FBS; Life Technologies, Carlsbad, CA, USA), 1% (*v*/*v*) penicillin/streptomycin (Life technologies, Carlsbad, CA, USA), 1.176 g/L of sodium bicarbonate (Sigma-Aldrich, Algés, Portugal), 4.76 g/L of 4-(2-Hydroxyethyl)piperazine-1-ethanesulfonic acid (HEPES) (Sigma-Aldrich, Algés, Portugal), 1 mL/L of Epidermal Growth Factor (EGF) and 1 mg/L of hydrocortisone > 98% (Sigma-Aldrich, Algés, Portugal). All experiments were performed between passages 4 and 9. Human dermal fibroblasts (HDF; ATCC, Barcelona, Spain) were cultured in Dulbecco’s modified Eagle’s medium (DMEM) supplemented with 10% (*v*/*v*) FBS (Life Technologies, Carlsbad, CA, USA) and 1% (*v*/*v*) penicillin/streptomycin (Life technologies, Carlsbad, CA, USA). All experiments were performed between passages 13 and 17. Cells were maintained at 37 °C in a humidified 5% CO_2_ atmosphere. Both human cells lines were purchased from ATCC, upon accord agreement. All procedures were performed in accordance with Good Laboratory Practice (Directive 2004/10/EC).

### 3.3. Viability and Apoptosis Evaluation by Flow Cytometry

To evaluate cell viability and apoptosis, HMEC-1 and HDF were treated with Cyanoflan at 0.25%, 0.50% and 0.75% (*w*/*v*), or left untreated (control), in incomplete cell culture media (without FBS supplement), for 24 h. Then, cells were washed with Phosphate Buffered Saline (PBS) and trypsinized for 5 min at 37 °C. After centrifugation (1200 rpm during 5 min), cell pellets were resuspended in Binding Buffer (Life technologies, Carlsbad, CA, USA). Then, each experimental condition was incubated with 5 µL of Fluorescein isothiocyanate (FITC)-conjugated Annexin V (Life technologies, Carlsbad, CA, USA) and 5 μL propidium iodide (PI, Life technologies, Carlsbad, CA, USA) for 10 min at room temperature protected from light. In addition, the cells were stained only with PI or Annexin V FITC (mono staining) or left unstained (negative control). 

Flow cytometry was performed using a BD Accuri C6 Plus Flow Cytometer (Becton-Dickinson, Franklin Lakes, NJ, USA), plotting at least 30,000 events for negative control and mono labels, and 50,000 events for double staining, after debris exclusion. Data was analyzed using FlowJo 10.7.1 software (Tree Star, Inc., Ashland, OR, USA).

### 3.4. Migration Analysis by Injury Assay

Cells were grown until 90% confluence. Using a pipette tip, cells were scrapped from the culture dish leaving a void space. Cells were then incubated for 16 or 24 h with distinct treatments: Cyanoflan at 0.25% and 0.50% (*w*/*v*) or left untreated (control). Cell migration to the damaged area was visualized and photographed on a phase contrast microscope (Nikon, Kingston upon Thames, UK), at a magnification of ×100, as previously reported [24]. Migration capacity was then quantified by measuring the migrated area over 24 h, using Image J. Results are expressed as percentage of injured area migrated in relation to the beginning (*t* = 0 h).

### 3.5. Reactive Oxygen Species (ROS) Formation

ROS generation in cell culture supernatant was performed using 2′,7′–dichlorofluorescin diacetate (DCFDA)-ROS detection kit (DCFDA Cellular ROS Detection Kit, Abcam, Cambridge, UK) according to manufacturer’s instructions. Briefly, HMEC-1 and HDF (3 × 10^4^ cells/well) were cultured in dark 96-well plates for 24 h. Then, cells were stained with 25 μM DCFDA followed by an incubation period of 45 min at 37 °C. Later, cells were treated with distinct treatments for 2 h. Fluorescence intensity (excitation: 485, emission: 535 nm) were quantified under a fluorescence microscope and ROS production was expressed as fluorescence units. Results are expressed as means ± standard error of the mean (SEM).

### 3.6. In Vivo Biocompatibility Assay 

Eighteen Wistar 8-week-old male rats (Charles-River, Barcelona, Spain) were sedated with isoflurane, shaved on the dorsum and the skin was cleaned with 70% ethanol. Two excisional wounds were created on dorsal midline using a biopsy punch (5 mm diameter), as previously described [25]. Then, animals were randomly divided into 3 experimental groups (*n* = 6), according to distinct experimental conditions: Cyanoflan at 0.25% and 0.5% (*w*/*v*) prepared in deionized water and reticulated with 1 M CaCl_2_ solution, or control (saline solution), and were applied topically every second day (50 μL per wound) for one week. After that, animals were sacrificed with isoflurane. Blood samples were collected, centrifuged (2000× *g* for 15 min) and used to evaluate systemic inflammatory and antioxidant markers. One skin wound and a liver biopsy of each animal were collected, fixed in 10% neutral-buffered formalin, processed by dehydration incubating through a graded series of ethanol, xylol and embedded in paraffin. Five µm-thick tissue sections were used for histological analysis. Skin tissue of the second wound of each animal were collected, snap-frozen and stored at −80 °C, to be used to quantify local inflammatory and antioxidant markers.

Animals were maintained under controlled conditions of temperature (23 ± 5 °C), humidity (35% ± 5%) and 12 h light/dark cycles, and access to diet and beverages were allowed ad libitum. All animal experiments were conducted at the animal house located at the Faculty of Medicine, University of Porto, and were carried out by trained technicians in accordance with the European Community policy for Experimental Animal Studies (European Community law dated from 24 November 1986 (86/609/CEE) with addendum from 18 June 2007 (2007/526/CE)).

After 21 days, all animal care and procedures were in accordance with the Portuguese Act 1005/92 (number 3, iii) and European Community guidelines (86/609/EEC) for the use of experimental animals.

### 3.7. Inflammatory Markers

#### 3.7.1. Interleukin-6 Quantification

The pro-inflammatory interleukin-6 was quantified to evaluate local and systemic inflammatory reaction. 50 mg of skin wounded tissue were homogenized with 2 mL of T-PER™ Tissue Protein Extraction Reagent (Merck, Darmstadt, Germany) with a MagNA Lyser (Roche, Amadora, Portugal) followed by protein content determination by Pierce™ Bicinchoninic acid (BCA) protein assay kit (Merck, Darmstadt, Germany). Subsequently, 50 µL of plasma or skin lysate (adjusted to protein content) were used to perform an ELISA assay to detect IL-6 (Sigma-Aldrich, Algés, Portugal), according to the manufacturer’s instructions. All samples were assayed in triplicate. Results were expressed as IL-6 concentration (pg/mL).

#### 3.7.2. N-acetyl-ß-D-glucosaminidase (NAG) Determination Assay 

N-acetyl-ß-D-glucosaminidase is a highly expressed enzyme in activated macrophages. To perform this assay, 100 µL of plasma was incubated with equal volume of the substrate p-nitrophenyl-N-acetyl-ß-D-glucosaminide solution at 37 °C. After 30 min, fresh 0.2 M glycine buffer (pH 10.6) was used as stop solution. Substrate hydrolysis was measured at 405 nm in a microplate reader (Thermo Electron Corporation, Multiskan Ascent), according to previous procedures [26]. All samples were assayed in triplicate. Results were expressed as NAG concentration (nmol/mL).

#### 3.7.3. Nitric Oxide (NO) Quantification

The quantification of total NO levels, including NO and their metabolites, nitrate and nitrite, was performed in the plasma using the Griess reagent method, as previously performed [26]. To perform this assay, 100 µL of plasma was incubated with equal volume of Griess reagent, for 15 min at room temperature. Then, the optical density was measured at 550 nm in a microplate reader (Thermo Electron Corporation, Multiskan Ascent). All samples were assayed in triplicate. Results were expressed as NO concentration (µM). 

#### 3.7.4. Superoxide Dismutase (SOD) Activity

The superoxide dismutase activity (inhibition rate %) was assayed in skin lysates by SOD determination kit (Sigma-Aldrich, Algés, Portugal) according to the manufacturer’s instructions. Briefly, sample solutions were mixed with water-soluble tetrazolium salt (WST) and enzyme working solutions followed by incubation at 37 °C for 20 min. Absorbance was recorded at 450 nm against blanks. Results were expressed as inhibition rate in comparison to control.

### 3.8. Statistical Analysis 

Statistical analysis was performed comparing the control group results with those of the different groups exposed to Cyanoflan at different concentrations, with one-way analysis of variance (ANOVA) multiple comparisons and Bonferroni post hoc test, using GraphPad Prism 8.0 software (GraphPad Software, San Diego, CA, USA). A two-way ANOVA, followed by pairwise comparisons with Bonferroni’s post-hoc test, was used to compare differences in viability and apoptosis, between control and Cyanoflan concentrations. Normality of data distribution was assessed using the Shapiro–Wilk test and for the homogeneity of variance with Levene’s test.

Data is expressed as mean ± SEM. Results were considered statistically significant whenever *p*-value ≤ 0.05.

## 4. Conclusions

In the present study, Cyanoflan, a naturally derived extracellular polymer, demonstrated the potential to be used as a novel skin dressing as it reveals biocompatibility, allowing the migration and proliferation of endothelial cells and fibroblasts. Moreover, cell viability remained closer to 100% without inducing apoptosis or necrosis, highlighting the optimal microenvironment for cell adhesion, proliferation and migration, which is crucial during skin wound healing. Accordingly, our in vivo results demonstrated that this biopolymer perfectly adapts to the wound bed without inducing systemic or local oxidative or inflammatory reaction. 

The functional properties of Cyanoflan suggest that it could be safely applied as a dressing for biomedical applications and/or used as a polymeric platform for the release of bioactive molecules that promote the healing process, therefore opening new possibilities for the development of therapeutical options designed to address the ever-increasing chronic and difficult-to-heal wounds, a huge clinical challenge.
